# The Practical Use of White Cell Inflammatory Biomarkers in Prediction of Postoperative Delirium after Cardiac Surgery

**DOI:** 10.3390/brainsci9110308

**Published:** 2019-11-02

**Authors:** Katarzyna Kotfis, Justyna Ślozowska, Krzysztof Safranow, Aleksandra Szylińska, Mariusz Listewnik

**Affiliations:** 1 Department of Anesthesiology, Intensive Therapy and Acute Intoxications, Pomeranian Medical University, 70-111 Szczecin, Poland; katarzyna.kotfis@pum.edu.pl (K.K.); justynagarlak@gmail.com (J.Ś.); 2 Department of Biochemistry and Medical Chemistry, Pomeranian Medical University, 71-111 Szczecin, Poland; chrissaf@mp.pl; 3 Department of Medical Rehabilitation and Clinical Physiotherapy, Pomeranian Medical University, 70-111 Szczecin, Poland; 4 Department of Cardiac Surgery, Pomeranian Medical University, 70-111 Szczecin, Poland; sindbaad@poczta.onet.pl

**Keywords:** POD, delirium, cardiac surgery, biomarkers, NLR, PWR, PLR, prediction index

## Abstract

Introduction: Postoperative delirium (POD) is associated with unfavorable outcomes. It may result from neuroinflammation and oxidative stress. The aim of this study was to evaluate the role of routinely available inflammatory markers derived from white blood cell count (WBC), for prognostic value in diagnosing delirium after cardiac surgery. Methods: We performed an analysis of data collected from patients undergoing planned coronary artery bypass grafting (CABG). Differential WBC and CRP (C-reactive protein) concentration were evaluated preoperatively (T0) and postoperatively at day 1 (T1), 3 (T3), 5 (T5) after CABG. Differences in neutrophil-to-lymphocyte ratio (NLR), platelet-to-lymphocyte ratio (PLR) and platelet-to-WBC ratio (PWR) between patients with (Del +) and without delirium (Del −) were evaluated. Patients were screened using CAM-ICU. Results: We included 968 patients in the study. Incidence of delirium was 13.3%. In the group with POD, the majority of patients were men (87/129, 67.44%), and the mean age was 72 years. Preoperative WBC (8.21 ± 3.04 G/l vs. 7.55 ± 1.86 G/l, *p* = 0.029) were higher and mean platelet count was lower (217.7 ± 69.07 G/l vs. 227.44 ± 59.31 G/l, *p* = 0.031) in patients with POD. Lower pre-operative PLR values (109.87 ± 46.38 vs. 120.36 ± 52.98, *p* = 0.026) and PWR values (27.69 ± 7.50 vs. 31.32 ± 9.88 *p* < 0.001) were found in patients with POD. Association was strongest for PWR and remained significant at T1 (*p* < 0.001), T3 (*p* < 0.001) and T5 (*p* < 0.001). Basing on coefficients of logistic regression a model for optimal prediction of POD was calculated: CARDEL Index (CARdiac DELirium Index) = 0.108 × Age + 0.341 × HBA1C − 0.049 × PWR with AUC of 0.742 (*p* < 0.001). Conclusions: The results of this study show that lower pre-operative levels of PLR and PWR were associated with POD after cardiac surgery. Pre-operative PWR showed strongest correlation with POD and may be a potential new biomarker associated with postoperative delirium. CARDEL prognosis index composed of age, HbA1c and PWR is good at predicting development of delirium after CABG.

## 1. Introduction

Delirium is defined as a disturbance of consciousness, presenting with a sudden onset, characterized by a fluctuating course of attention and accompanied by a change in cognition or perception [1]. It is an acute neuropsychiatric syndrome that impairs the ability of the patient to receive information, process it and store for further recall, that requires monitoring and treatment [2]. Reported to be a relatively common complication among patients undergoing cardiac surgery, it is associated with increased mortality, longer intensive care unit (ICU) and hospital stay, loss of independence, and an increased risk of developing postoperative cognitive dysfunction [3,4,5,6,7]. A new classification of delirium phenotypes is based on clinical risk factors and includes sedative-associated, sepsis-associated, hypoxic and metabolic delirium [8]. This emphasizes that delirium is a cumulative effect of multiple interconnecting insults, that include neuroinflammation and oxidative stress [9,10]. Most likely, the cascade of events starts with generalized inflammation leading to endothelial dysfunction that increases the permeability of the blood-brain barrier and the development of inflammatory changes of the nervous tissue, damage to neurons and exaggerated response of microglial cells [10]. 

The diagnosis of delirium is based upon clinical observation therefore it should involve validated bedside psychometric diagnostic tools for accuracy—either CAM-ICU (Cognitive Assessment Method for ICU) or ICDSC (Intensive Care Delirium Screening Checklist) [2]. Despite active monitoring there are a number of patients that remain undiagnosed, with either hypoactive or subsyndromal delirium [11,12]. Research involving biomarkers did not indicate any single particular one for delirium screening, rather an identification of a panel of biomarkers that may lead to an accurate and timely diagnosis and improvement in prediction and recognition of delirium [13,14,15]. Therefore, the researchers keep searching for an accurate ideal biomarker for delirium, that would have a high predictive value, be readily available, reliable and cheap. 

The differential white blood cell (WBC) count is one of the tests routinely performed in the majority of hospitalized patients at no additional cost. The number of information provided by it, both in health and in disease, is larger that is commonly thought and cannot be overestimated. Transient elevations of the serum white blood cell count and decreases of platelet count are regarded as normal physiological responses to inflammation. Both, the neutrophil-to-lymphocyte ratio (NLR), and the platelet-to-lymphocyte ratio (PLR), as well as platelet-to-WBC ratio are easily available markers of generalized inflammation reported in different research settings [16,17]. An increase in the NLR level has been identified as an outcome measure in cerebrovascular and cardiovascular diseases [18,19], neuropsychiatric disorders (i.e., stroke, Alzheimer’s disease or schizophrenia) [16,20,21], autoimmune diseases (i.e., systemic lupus erythematosus, ulcerative colitis) [22,23] and various malignant tumors [24,25]. A pilot study performed by Egberts et al. reported an association between increased NLR and delirium in elderly patients admitted to a geriatric unit [17].

Some studies have shown that in certain medical conditions a better predictor of inflammation is the platelet-to-lymphocyte ratio—this was found in chronic renal failure, autoimmune diseases, and cardiovascular diseases [26,27]. Serum inflammatory markers based on the differential WBC count, NLR and PLR, have been found to be better predicting factors for mortality and outcome in various medical conditions, as well as predictors of cardiovascular risk as compared with traditional infection markers, including C-reactive protein (CRP) or the total leucocyte count [28,29]. 

To date, a possible association between the panel of white-cell derived biomarkers and postoperative delirium has not been investigated. Therefore, the authors aimed at evaluating which of the markers of inflammation derived from the white cell count, namely NLR, PLR and platelet-to-WBC count ratio (PWR), as well as CRP could serve best for their prognostic value before and after the operation in prediction of delirium after cardiac surgery. Moreover, the aim of this study was to create a model that, using objective clinical and laboratory data, would be useful in assessing the risk of delirium after CABG (coronary artery bypass grafting).

## 2. Methods

### 2.1. Study Design and Patient Selection

A retrospective cohort analysis was performed including data collected from all patients undergoing cardiac surgery in a cardiac surgery department at the university hospital between 1 January 2014 and 31 December 2016. From the total group of patients undergoing the coronary artery bypass grafting procedure (CABG) at our institution, we included data regarding only patients undergoing planned, isolated CABG. We excluded patients with known pre-operative delirium or cognitive disorder (MMSE below 24 points, known dementia or mild cognitive impairment, MCI), neuropsychiatric disorder (i.e., cognitive impairment, depression, schizophrenia, epilepsy), with known pre-operative infection or who developed a significant postoperative infection (pulmonary, urinary, sepsis) within the first 48 h after CABG. Patients who died either during or within first 24 h after the operation were excluded from further analysis. Only patients with a full set of blood results were included in the analysis. We divided the patients into two groups based on the presence (Delirium +) or absence (Delirium −) of delirium in the postoperative period. 

**Figure 1:**
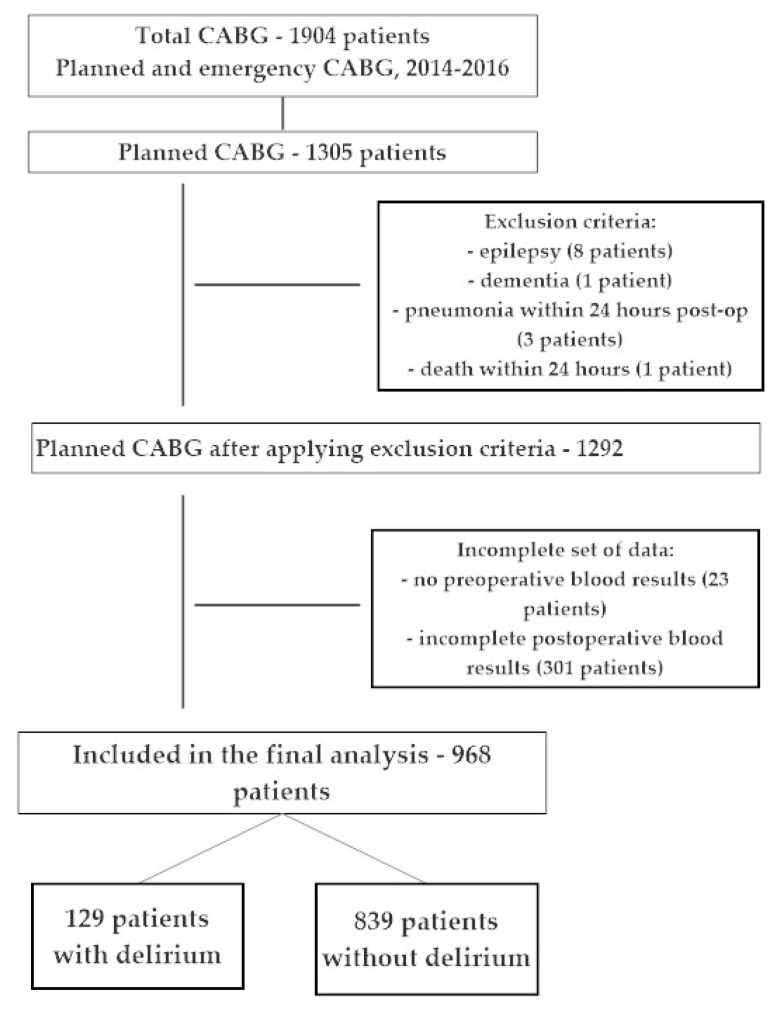
Study flowchart.

Figure 1 shows that from a total group of 1904 patients undergoing general anesthesia for coronary artery bypass surgery (CABG) between 2014 and 2016, 1305 patients underwent a planned, isolated CABG procedures, which created a very homogeneous group of patients. The study excluded patients with epilepsy (8), with dementia (1), patients who died during the first 24 h after surgery (1), patients with pneumonia within 24 h after surgery (3) and 23 patients who did not have pre-operative laboratory tests. After applying the exclusion criteria, the final analysis included 968 patients with complete perioperative data. 

### 2.2. Data Collection

We reviewed the medical records for demographic data and comorbidities, followed by the preoperative anesthetic visit with a detailed questionnaire. Concomitant diseases included: cardiovascular—atrial fibrillation, congestive heart failure (only NYHA classes III and IV were recorded), hypertension, myocardial infarction, internal carotid artery stenosis and extracardiac arteriopathy; metabolic—thyroid disorders, dyslipidemia, impaired glucose tolerance, diabetes; pulmonary—chronic obstructive pulmonary disease; renal—acute kidney injury and chronic renal insufficiency; neurological—stroke, transient ischemic attack (TIA). Data regarding smoking prior to the operation was also recorded. We used EuroScore Logistics 2 scale to calculate the perioperative risk for each patient. 

We collected data regarding the postoperative outcome, which included the following information: intubation time, hospital length of stay, ICU length of stay, 30-day and 1-year mortality, as well as postoperative complications (cardiac and pulmonary, neurological, renal, infectious) using chart review. All laboratory testing was performed as part of routine preoperative and postoperative care. All biomarkers were collected in the morning, by the clinical staff, transported to the central laboratory immediately after collection and processed in the clinical lab.

Both pre-operative evaluation in the cardiac surgery outpatient clinic and premedication anesthetic assessment was performed to ensure patient safety. Intraoperative surgical and anesthetic approach followed a well-established local protocol. All patients were anesthetized with intravenous induction of general anaesthesia using fentanyl and etomidate, followed by pancuronium for muscle relaxation to facilitate tracheal intubation. For maintenance of general anesthesia an inhalational agent (sevoflurane) was used along with additional doses of fentanyl for pain control and pancuronium for muscle relaxation. Heparin was administered intravenously before initiation of the cardio-pulmonary bypass (CPB) and was guided by ACT (activated clotting time). Data regarding the procedure included the following information: operating time, cardiopulmonary bypass time, cross-clamping time and the volume of postoperative drainage. 

After the operation and after weaning the patient from CPB, protamine sulphate was used to reverse the action of heparin guided by ACT. After the operation patients were transferred to cardiac ICU and remained intubated and mechanically ventilated as long as they required postoperative monitoring and treatment. Postoperative pain was controlled with intravenous morphine infusion and non-opioid analgesic administration (paracetamol or metamizole). Postoperatively patients were sedated with either propofol or dexmedetomidine, pain control was provided by multimodal analgesia (continuous intravenous morphine and non-opioid analgesic medications). After meeting extubation protocol criteria patients were extubated and transferred to the cardiac surgery ward. 

### 2.3. Ethical Consideration

According to the Declaration of Helsinki this study has been submitted to the bioethical committee and received a waiver due to the retrospective character (Bioethical Committee of Pomeranian Medical University in Szczecin, Poland, decision no. KB-0012/257/06/18, 28.06.2018). Prior to the operation each patient signed a written informed consent for both surgery and anesthesia with a consent for research data collection. All analyzed data was anonymous to ensure confidentiality. 

### 2.4. White Cell Biomarkers and CRP Analysis

The WBC counts and CRP level were evaluated as part of routine perioperative testing. The blood was collected preoperatively (time T0, i.e., blood collected at admission, 24 h prior to surgery) and postoperatively (time T1, T3, T5 at day 1, 3, 5) after CABG. Blood morphology with full leukocyte differentiation was determined using the Sysmex XN-2000 analyzer the FSC, SSC DIFF, impedance, and spectrophotometric methods with sodium lauryl sulfate were used to determine hemoglobin. The C-reactive protein level was determined by the immunoturbidimetric method using a Roche Cobas 8000 analyzer. We calculated the neutrophil-to-lymphocyte ratio by dividing the absolute neutrophil count by the absolute lymphocyte count. The platelet-to-lymphocyte ratio was calculated by dividing the absolute platelet count over the absolute lymphocyte count. We calculated platelet-to-WBC ratio by dividing by the absolute platelet count over the WBC.

### 2.5. Delirium Assesment

We used the Polish version of the CAM-ICU test to screen all patients for delirium in the cardiac -ICU and in the post-operative ward [6]. CAM-ICU was performed twice a day (morning and evening) during the first 6 days of the postoperative course. The team of nurses, anesthesiologists, intensivists and cardiac surgeons were involved in this process. The final delirium diagnosis was made by consultant neurologist using the standard criteria of Diagnostic Statistical Manual of Mental Disorders, fifth edition [1]. The initial delirium screening was done in sedated patients in the cardiac-ICU by a nurse and a doctor (either of the above). Subsequent observations were carried out in all patients after they have been extubated (usually within 12 h after CABG). 

### 2.6. Statistical Analysis 

In order to characterize the population of this study we used descriptive statistics. We used proportions to present categorical variables and Chi-square test for comparison between the groups. Continuous variables were presented as means with standard deviation. We used Mann–Whitney *U*-test to compare baseline characteristics between patients with and without delirium. Spearman’s correlation coefficient was used to analyze the correlations between quantitative variables. In the next step, we used multivariate logistic regression analysis to find parameters most strongly and independently related to the occurrence of delirium. The multivariate models were constructed by forward selection of independent variables with two different Wald p-values required for inclusion. The “exploratory” model used classical *p* < 0.05 threshold, while the “reliable” model used *p* < 0.001 threshold. The lower threshold was applied as a correction for multiple comparisons, to prevent inclusion of variables only randomly associated with delirium into the reliable predictive model (nearly 50 independent pre-operative variables were analyzed as potential predictors of delirium, so the Bonferroni-corrected p is 0.05/50 = 0.001). Based on the reliable multivariate model parameters and depending on the contribution of each analyzed variable a formula for optimal prediction of delirium was calculated and presented as CARDEL Index (CARdiac DELirium Index). We also performed a receiver operating characteristic (ROC) analysis to determine the diagnostic value of the CARDEL index and its components for predicting the delirium. The cut-off points, which maximized Youden index (sensitivity + specificity − 1) were presented. Statistical significance was determined as *p* value below 0.05. The data was analyzed using Statistica 13 software with Medical Bundle 4.0.

## 3. Results

### 3.1. Baseline Characteristics

The baseline characteristics of the study group are depicted in Table 1. Out of the whole study cohort 13.3% (129/968) had delirium during the first 6 days after CABG. In the group with delirium majority of patients were men (87/129, 67.44%), they were older with mean age of 71.69 ± 7.96 years (*p* = 8.06) and smoking was less common in this group (10.08% vs. 15.38%, *p* = 0.07). Baseline laboratory values showed significant differences between the two groups, with higher pre-operative glycated hemoglobin level (6.38 ± 1.18 vs. 6.06 ± 1.02, *p* < 0.001) and higher creatinine level (1.11 ± 0.79 vs. 0.97 ± 0.52, *p* < 0.001).

Patients with delirium more often suffered from hypertension (83.72% vs. 74.26%, *p* = 0.026), significant internal carotid artery stenosis (14.73% vs. 6.08%, *p* < 0.001), extracardiac atherosclerosis (29.46% vs. 15.97%, *p* < 0.001), COPD (8.53% vs. 4.05%, *p* = 0.043) and chronic renal failure (12.40% vs. 5.84%, *p* = 0.009), as depicted in Table 2.

Table 3 shows outcome data for both subgroups. Patients with delirium had longer time of postoperative mechanical ventilation (815.04 ± 584.10 min vs. 735.83 ± 878.28 min, *p* = 0.005) and longer hospitalization time (11.37 ± 13.36 days vs. 7.99 ± 4.17 days, *p* < 0.001). Mortality in the group with delirium was significantly higher, both at 30 days (4.65% vs. 1.07%, *p* = 0.007) and at 1 year after surgery (12.40% vs. 4.77%, *p* = 0.001).

### 3.2. Analysis of Inflammatory Markers

Table 4 presents mean levels of pre-operative inflammatory markers in patients from both subgroups. Higher mean levels of leucocytes (8.21 ± 3.04 vs. 7.55 ± 1.86, *p* = 0.029) and CRP (6.33 ± 12.34 vs. 4.06 ± 7.80, *p* = 0.015) were observed in delirious patients, along with lower mean levels of thrombocytes (217.7 ± 69.07 vs. 227.44 ± 59.31, *p* = 0.031). When analyzing derived parameters, we noted that the p-value for difference of NLR between delirious and non-delirious patients was not significant (*p* = 0.628). 

Lower pre-operative PLR values (109.87 ± 46.38 vs. 120.36 ± 52.98, *p* = 0.026) and PWR values (27.69 ± 7.50 vs. 31.32 ± 9.88, *p* < 0.001) were found in patients with postoperative delirium. 

**Figure 2:**
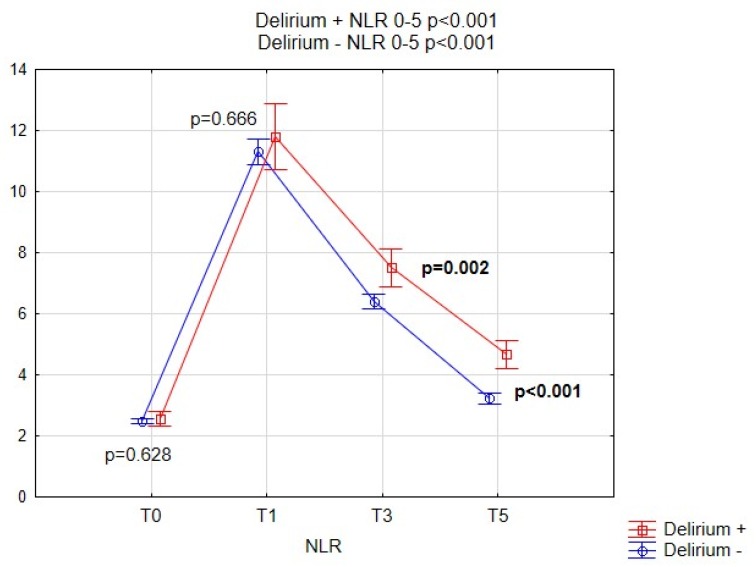
Pre-operative and post-operative NLR values for patients with and without POD. NLR, neutrophil-to-lymphocyte ratio; POD, Postoperative delirium.

**Figure 3:**
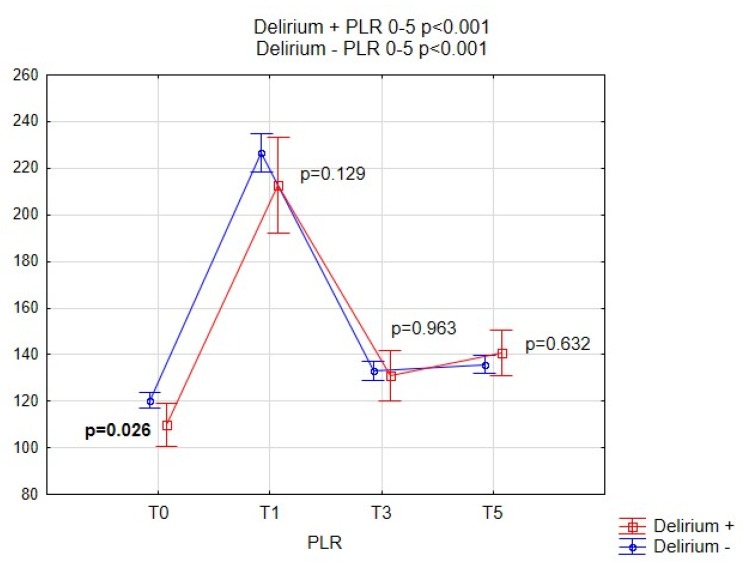
Pre-operative and post-operative PLR values for patients with and without POD. PLR, platelet-to-lymphocyte ratio; POD, Postoperative delirium.

**Figure 4:**
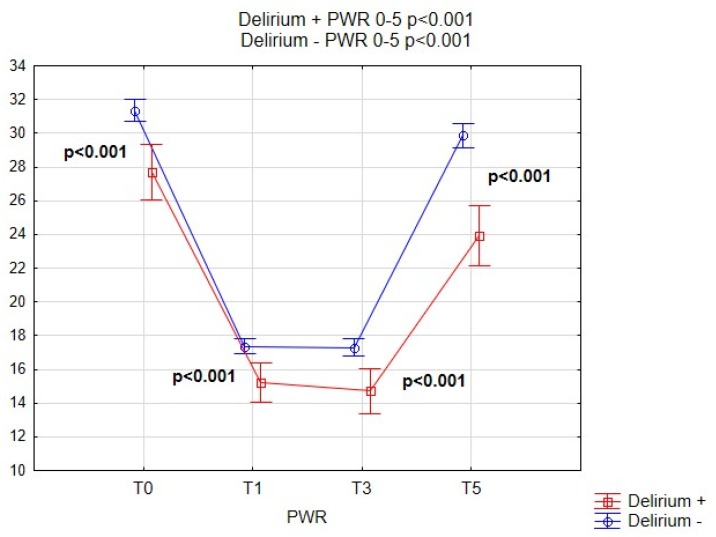
Pre-operative and post-operative PWR values for patients with and without POD. PWR, platelet-to-WBC ratio; POD, Postoperative delirium.

**Figure 5:**
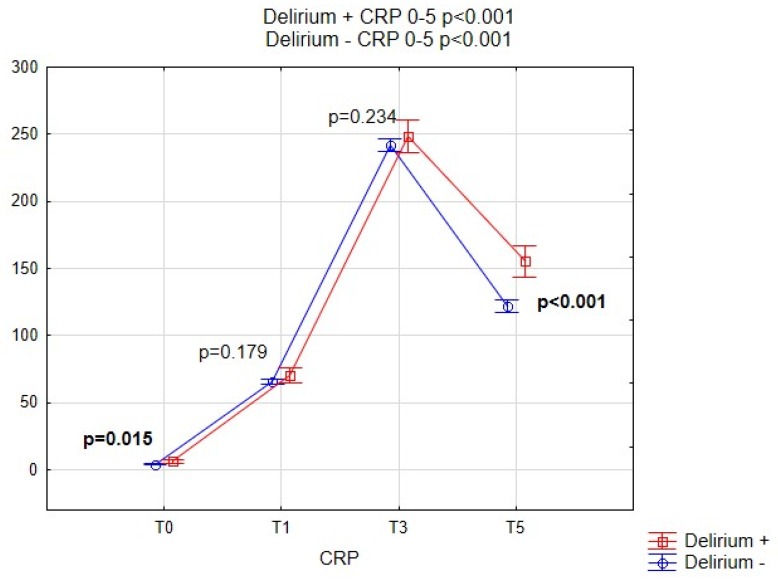
Pre-operative and post-operative CRP values for patients with and without POD. CRP, C-reactive protein; POD, Postoperative delirium.

Figure 2, Figure 3, Figure 4 and Figure 5 show the performance of NLR, PLR, PWR and CRP before and after the operation in both subgroups of patients. Comparison of selected indicators between patients with delirium and without delirium showed statistically significant differences (*p* < 0.001). The results are presented on individual figures.

After the operation at day 1 only the platelet count was significantly lower in patients with delirium (168.56 ± 63.38 vs. 176.63 ± 49.75, *p* = 0.019), therefore the PWR was also significantly lower (15.23 ± 5.21 vs. 17.36 ± 7.04, *p* < 0.001). The association was strongest for PWR and remained significant on day one (T1, *p* < 0.001), day three (T3, *p* < 0.001) and day five (T5, *p* < 0.001). Data in Table 5 shows detailed post-operative laboratory values for patients from both groups.

The pre-operative value of the CRP differed significantly between patients with and without delirium (*p* = 0.015), with levels 6.33 ± 12.34 vs. 4.06 ± 7.80, respectively. In the post-operative period, the CRP was greatly elevated, yet the differences between the two sub-groups showed no statistical significance on day 1 (T1, 70.26 ± 34.39 vs. 65.63 ± 32.12, *p* = 0.179) on day 3 (T3, 248.13 ± 68.45 vs. 241.61 ± 70.95, *p* = 0.234) after CABG. The differences on day 5 were once again significant (T5, 155.29 ± 73.56 vs. 122.09 ± 66.19, *p* < 0.001). 

### 3.3. Development of CARDEL Index

Univariate analysis showed significant associations with the occurrence of delirium for many pre-operative variables, with older age having the highest discriminative value. In order to investigate which variables are the strongest and independent factors allowing to predict delirium after cardiac surgery, a multivariate logistic regression analysis was performed in which the dependent variable was delirium. Data on comorbidities and additional pre-operative data, including blood morphology parameters, for which univariate analysis showed the strongest association with the occurrence of delirium after surgery, were included as independent variables in the successive multivariate models constructed by forward selection. Multivariate logistic regression showed that most of risk factors for delirium demonstrated by univariate analysis are not independent risk factors (e.g., GFR (glomerular filtration rate)).

An exploratory model analyzing sex, age, NYHA, occurrence of peripheral atherosclerosis, HbA1C and PWR as independent variables associated with delirium as a dependent variable showed that all variables are independently (*p* < 0.05) associated with the occurrence of delirium. This model shows that men have smaller odds of experiencing delirium by 45%. With each subsequent year of life, the chance of experiencing a delirium is higher by 11%. The higher the value in the NYHA class, the greater the chance of experiencing delirium (by 35.5% per class). The occurrence of peripheral atherosclerosis is associated with an almost twice increased odds of delirium. HbA1C higher by 1% is associated with greater odds of developing a delirium by 33%. The PWR value higher by one unit reduces the odds of delirium by 5%. Detailed data is presented in Table 6. 

One of the aims of this study was to create a reliable model that, using objective pre-operative clinical and laboratory data, will be useful in assessing the risk of delirium after CABG. Then the logistic regression model was created with forward selection at stringent (*p* < 0.001) significance threshold criterion, addressing the issue of multiple comparisons, containing only three independent variables most strongly associated with delirium: age, HbA1c and PWR (Table 7). It is worth noting that the three independent variables included in the model are completely uncorrelated and therefore completely independent of each other (absolute values of Spearman rank correlation coefficients are below 0.04 and variance inflation factor (VIF) values are below 1.01).

Basing on the coefficients of the reliable logistic model, an index, called CARDEL, consisting of age, HbA1c and PWR was calculated as an optimal predictor of delirium:

CARDEL Index = 0.108 × Age + 0.341 × HBA1C − 0.049 × PWR

**Figure 6:**
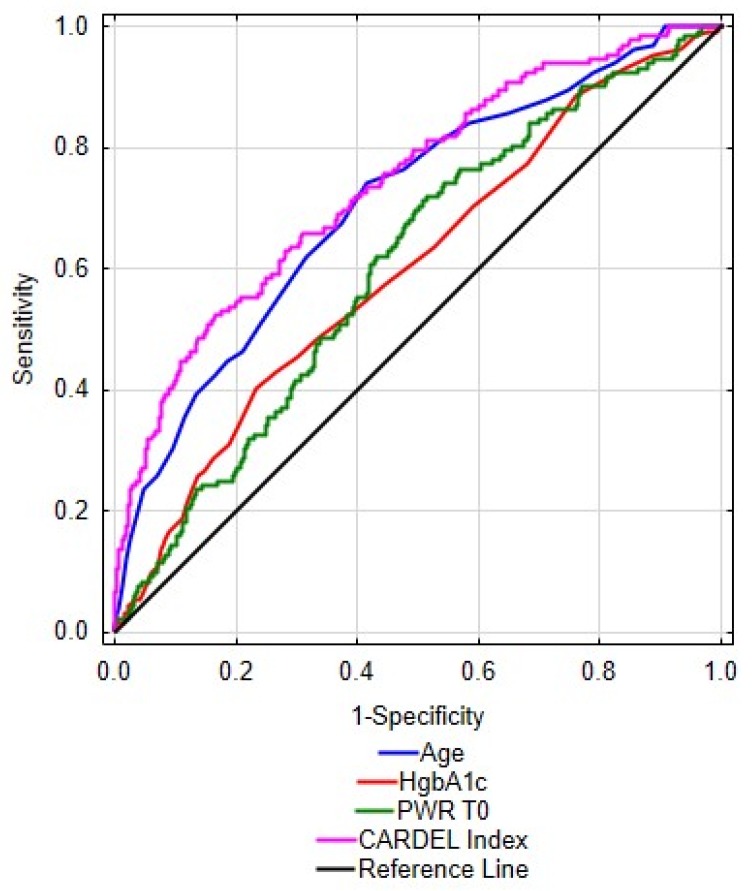
Receiver operating characteristic (ROC) analysis for the CARDEL index as a determinant of postoperative delirium after CABG compared to the separate components of the index itself. Legend: AUC—area under the curve, HbA1c—glycated hemoglobin, PWR—P Platelet-to-WBC Ratio.

The goodness-of-fit measure of the CARDEL model, Nagelkerke pseudo *R*^2^, was 0.182, and the value of likelihood ratio (LR) was 101.1 (*p* < 0.000001). The ROC analysis showed that the CARDEL index is better at predicting the development of delirium after CABG than any of these factors alone, as shown in Figure 6.

The data in Table 8 shows that the combined CARDEL index has the highest area under the ROC curve (AUC)—0.742 with *p* < 0.001. Presented exemplary cut-off points are based on maximization of Youden index, which is equivalent to maximization of the sum of sensitivity and specificity of each predictor. For CARDEL the cut-off value was estimated at 8.26 with sensitivity at 0.612 and specificity at 0.753.

## 4. Discussion

In this analysis of a large database including a homogenous group of patients undergoing CABG, we identified the platelet-to-WBC ratio (PWR) as a novel and independent predictor of postoperative delirium in patients undergoing planned cardiac surgery. A lower Platelet/WBC ratio was independently associated with a higher risk of postoperative delirium in patients undergoing CABG. To our knowledge, this is the first study to identify the Platelet-to-WBC ratio as a predictor of postoperative delirium after cardiac surgery. The mechanistic relationship between the PWR and delirium remains to be elucidated. The pre-operative level of white blood cells, along with the platelet level and their derived ratios may be used as surrogates for the baseline health status of patients in the pre-operative period. Those levels and ratios differ in patients who develop post-operative delirium due to neuroinflammation or immune system imbalance.

Not enough is known regarding the pathogenesis of delirium [30]. Further identification of biomarkers may be helpful in broader understanding of pathogenic mechanisms, prognosis and diagnosis of delirium. The delirium markers identified so far point to inflammation and oxidative stress as the underlying mechanisms of delirium pathogenesis [9,10,31]. The identification of known markers is expensive and time consuming, therefore it serves research purposes rather than being used in everyday clinical practice. An ideal biomarker should be cheap, repeatable, easily available, highly sensitive and very specific. The hematology parameters – differential white cell count seem to meet most of the above-mentioned criteria for an ideal diagnostic or prognostic marker. Numerous studies have shown the relationship between elevated NLR and cerebrovascular diseases [21], schizophrenia [16], Alzheimer’s disease, disease severity and poor prognosis in cardiovascular diseases [19] or cancer [24,25]. 

The response of the immune system induced by surgical stress leads to an increase in the neutrophil count accompanied by the concomittant decrease in the lymphocyte count, along with a decrease in platelet count [32,33]. It has been shown that the composition of peripheral blood cells has been associated with the risk of post-operative complications in patients undergoing high-risk vascular procedures [34]. When considering delirium, a certain degree of neuroinflammation may be seen as elevation in the level of neutrophils and the neutrophil-to-lymphocyte ratio [35]. Moreover, a study performed by Egberts et al. pointed to the potential relationship between impaired NLR and delirium in the elderly population [17]. The NLR level has been proposed as a novel biomarker in various inflammatory or metabolic diseases (i.e., SLE, ulcerative colitis, inflammatory arthritis, diabetes mellitus, coronary artery disease), but its value has not been proven in patients with delirium in our study [36]. 

Some studies indicate a superior role of PLR over NLR [37], very numerous studies dispute the role of the commonest derived parameter—the platelet-to-white blood cell ratio—calculated from blood morphology, without the need to perform the differential white cells analysis, which makes it very inexpensive and easy to obtain. Our study has shown that that lower pre-operative mean PLR values and lower PWR values were found in patients with postoperative delirium. The association was strongest for PWR and remained significant at T1 (*p* < 0.001), T3 (*p* < 0.001) and T5 (*p* < 0.001). PWR has recently been shown to be an independent prognostic predictor for outcomes in some diseases. According to Chen et al. the platelet-to-WBC ratio on admission to the hospital may be useful at predicting the 90-day outcome in patients with ischemic stroke who received intravenous thrombolysis [38]. It was also useful in predicting post-operative infectious complications after radical nephrectomy in patients with renal cancer [39]. 

The mechanisms of neuroinflammation in patients undergoing cardio-pulmonary bypass have been described previously and the role of neutrophils in this process must be acknowledged to better understand the mechanisms leading to changes in serum NLR and PWR. We hypothesize that changes in the white blood cells differential count, as well as the CRP concentration may show that post-cardiac surgery delirium is associated with inadequate immune system response. Growing evidence suggests that neutrophils and lymphocytes are major effectors of acute inflammation, including neuroinflammation in delirium among elderly patients and post-stroke delirium [17,40]. The non-specific immune system activation, depicted by an increase in the neutrophil count and a decrease in the lymphocyte count, may be the first line response during generalized stress, leading to an imbalance between neutrophils and lymphocytes in the peripheral circulation [41,42]. The degree of neutrophil count elevation and the subsequent NLR rise may depict the degree of neuroinflammation. This observation may be the basis of delirium prediction by systemic inflammatory indicators [35]. Lymphopenia has been described as a known predictor of mortality in critically ill patients and may be regarded as a bedside marker of immunosuppression [33,35]. Inoue et al. have undertaken a study to determine whether lymphopenia was associated with ICU delirium. These researchers reported that patients with decreased lymphocyte levels showed a trend towards a higher chance of ICU delirium (*p* = 0.07) [30]. An imbalance of derived inflammatory white blood cell factors (NLR, PWR) may reflect subclinical inflammation [33]. 

One of the aims of the study performed by us was to create a model that, using objective pre-operative clinical and laboratory data, would be useful in assessing the risk of delirium after CABG. The index being a function of age, HbA1c and PWR was analyzed as a CARDEL index to check its diagnostic efficiency. In addition, it was checked whether correlations exist between the above three variables. Variables included in the model are completely uncorrelated and therefore completely independent of each other, therefore the index CARDEL can be treated as an indicator for delirium prediction. The analysis showed that the CARDEL index is better in predicting the development of delirium after CABG than any of these factors alone. An important virtue of this model is the use of information regarding the components of the immune system and preoperative laboratory values, because it has been suggested that the pathophysiology of delirium is multifactorial. The CARDEL index is simple to count, easy to analyze and, above all, cheap, because it was calculated using routine, easily accessible and objective pre-operative parameters. However, it must be underlined that this is a preliminary analysis of the CARDEL index and further research must follow to confirm these results. We plan to perform an independent prospective observational study in a different patient population to further investigate the results obtained in this study. 

No other research reported a predictive model based on routine pre-operative values for predicting the occurrence of delirium after cardiac surgery. A systematic analysis of the studies involving predictive models for delirium in the elderly population in the hospital was carried out by Lindroth et al. [43] Their analysis covered information retrieved from accessible databases (CINAHL, Cochrane, Embase PubMed, PsychINFO, SocinFO, Web of Science) from 1 January 1990 until 31 December 2016 and included studies involving patients over 60 years old, hospitalized, in which the authors developed and approved a prognostic prediction model for delirium. A priori analyzes excluded patients with delirium associated with alcohol and analyzes involving less than 50 people. After a complete review of 192 studies, 27 studies were included in the final analysis. Twenty-three forecasting models of delirium were identified. The assessment of delirium was usually unsystematic, which resulted in a varied incidence of delirium in the populations studied. Fourteen models have been externally validated with ROC AUC between 0.52 and 0.94. The authors identified design constraints, data collection methods and model metrics reporting statistics, and concluded in their summary that delirium prediction models, exhibit variable and inadequate prediction capabilities [43]. Lindroth et al. emphasized the need to develop robust models for predicting delirium in hospitalized patients to create predictive systems for specific populations [43]. A recent prospective observational study called DELIAS Study was performed by Kotfis et al. in patients with acute ischemic stroke, with an aim to assess whether the ratio of neutrophil-to-lymphocyte count can be used as a potential prognostic marker for delirium in patients with ischemic stroke [40]. As a result of various combinations of laboratory inflammatory markers and clinical parameters, the authors proposed the DELIAS index based on logistic regression, with the area under the ROC curve of 0.801 and *p* < 0.001). The authors concluded that NLR can be considered as a potential predictor of delirium after acute ischemic stroke, and the DELIAS index, easily calculated on the basis of combined laboratory and clinical parameters, shows the highest predictive value for delirium in the analyzed group of patients presenting with acute ischemic stroke [40]. 

This study exhibits certain limitations. First of all, this study is a single-center analysis and further studies are necessary to confirm our results in a different population of patients. Second, this was a retrospective analysis, therefore the data available may be limited and may not include all information. Third, the observational design of the study may limit the ability to identify all causal associations. Fourth, certain co-morbidities might influence the mean levels of white cell subtypes although patients with chronic inflammation and inflammatory diseases were excluded from the analysis. Fifth, delirium assessment was completed by routine clinical staff and it is well documented that clinical staff routinely miss delirium, especially hypoactive delirium.

A definite strength of this analysis is a very large number of patients included. This provides reliable data about easy to obtain and calculate biomarkers. The value of white cell count markers cannot be overestimated in patients undergoing surgery, as this laboratory workup is available from almost every hospitalized patient. Another important factor is the availability of serial measurements of NLR, PLR and PWR based on white cell count as this is easily obtainable at pre-defined time-points before and after the surgery. 

## 5. Conclusions

We conclude that lower levels of PLR and PWR were associated with POD after cardiac surgery, along with increased levels of CRP. We observed no significant difference for the NLR between patients with and without delirium. Preoperative PWR showed strongest correlation with POD and may be a potential new biomarker of neuroinflammation associated with delirium. The CARDEL prognosis index composed of age, HbA1c and PWR is better at predicting the development of delirium after CABG than any of these factors alone. The CARDEL index is a simple to calculate, easy to analyze and, above all, a cheap indicator, because it uses readily available laboratory values performed routinely and objective pre-operative parameters.

## Figures and Tables

**Table 1:** Baseline characteristics and perioperative data for patients with and without Postoperative delirium (POD) after coronary artery bypass grafting (CABG). POD, Postoperative delirium; CABG, coronary artery bypass grafting.

Variables	Delirium (+) (*n* = 129)	Delirium (−) (*n* = 839)	*p*-Value
Demographic data			
Age (years), mean ± SD	71.69 ± 7.96	65.37 ± 7.77	<0.001
Sex, male, *n* (%)	87 (67.44)	654 (77.95)	0.012
BMI (kg/m^2^), mean ± SD	28.74 ± 4.03	29.02 ± 4.36	0.424
Smoking, *n* (%)	13 (10.08)	129 (15.38)	0.070
Perioperative risk			
ESL2 (%), mean ± SD	2.74 ± 2.14	1. 84 ± 1.78	<0.001
Pre-operative data			
Hemoglobin, mean ± SD	8.45 ± 0.86	8.59 ± 0.85	0.03
CKMB, mean ± SD	31.15 ± 41.06	25.70 ± 33.13	0.06
HbA1c, mean ± SD	6.38 ± 1.18	6.06 ± 1.02	<0.001
Creatinine, mean ± SD	1.11 ± 0.79	0.97 ± 0.52	<0.001
GFR (mL/min/1.73m^2^), mean ± SD	69.88 ± 20.67	81.15 ± 18.39	<0.001

Legend: BMI—Body Mass Index, EF—ejection fraction, ESL—EuroScore Logistic 2, *n*—number of patients, NYHA—New York Heart Association, GFR—glomerular filtration rate.

**Table 2:** Co-morbidities in both study groups with delirium and without POD after CABG. POD, Postoperative delirium; CABG, coronary artery bypass grafting.

Variable	Delirium (+) (*n* = 129)	Delirium (−) (*n* = 839)	*p*-Value
Concomittant diseases			
Arterial hypertension, *n* (%)	108 (83.72)	623 (74.26)	0.026
Myocardial infarction, *n* (%)	79 (61.24)	433 (51.61)	0.051
STEMI, *n* (%)	44 (34.11)	258 (30.75)	0.506
NSTEMI, *n* (%)	38 (29.46)	193 (23.00)	0.136
AF paroxysmal, *n* (%)	10 (7.75)	54 (6.44)	0.712
AF persistent, *n* (%)	7 (5.43)	33 (3.93)	0.578
Diabetes (on insulin), *n* (%)	20 (15.5)	95 (11.32)	0.222
Diabetes (oral medications), *n* (%)	56 (43.41)	291 (34.68)	0.068
Impaired glucose tolerance, n (%)	2 (1.55)	32 (3.82)	0.297
Stroke with paresis, *n* (%)	3 (2.33)	19 (2.26)	0.784
Stroke without paresis, *n* (%)	5 (3.88)	17 (2.03)	0.319
TIA, *n* (%)	3 (2.33)	6 (0.72)	0.199
ICA stenosis, *n* (%)	19 (14.73)	51 (6.08)	<0.001
Extracardiac atherosclerosis, *n* (%)	38 (29.46)	134 (15.97)	<0.001
COPD, *n* (%)	11 (8.53)	34 (4.05)	0.043
Chronic renal failure, *n* (%)	16 (12.40)	49 (5.84)	0.009
Acute renal failure, *n* (%)	2 (1.55)	2 (0.24)	0.154
Dialysis, *n* (%)	1 (0.78)	7 (0.83)	0.650

Legend: AF—atrial fibrillation, COPD—chronic obstructive pulmonary disease, ICA—internal carotid artery, *n*—number of patients, NSTEMI—non-ST-elevation myocardial infarction, STEMI—ST-elevation myocardial infarction, TIA—transient ischemic attack.

**Table 3:** Outcome data for patients with and without POD after CABG. POD, Postoperative delirium; CABG, coronary artery bypass grafting.

Variables	Delirium (+) (*n* = 129)	Delirium (−) (*n* = 839)	*p*-Value
Mechanical ventilation (min), mean ± SD	815.04 ± 584.10	735.83 ± 878.28	0.005
Hospital length of stay (days), mean ± SD	11.37 ± 13.36	7.99 ± 4.17	<0.001
Mortality at 30 days, *n* (%)	6 (4.65)	9 (1.07)	0.007
Mortality at 1 year, *n* (%)	16 (12.40)	40 (4.77)	0.001

Legend: *n*—number of patients, SD—standard deviation.

**Table 4:** Mean levels of pre-operative inflammatory markers and derived parameters for patients with and without POD after CABG. POD, Postoperative delirium; CABG, coronary artery bypass grafting.

	Delirium + (*n* = 129)	Delirium – (*n* = 839)	*p*-Value
**Inflammatory parameters—DAY 0**			
Total WBC count (×10^9^/L), mean ± SD	8.21 ± 3.04	7.55 ± 1.86	0.029
Lymphocyte count (×10^9^/L), mean ± SD	2.37 ± 2.03	2.09 ± 0.71	0.499
Neutrophil count (×10^9^/L), mean ± SD	4.96 ± 1.83	4.64 ± 1.54	0.098
Platelets (×10^9^/L), mean ± SD	217.7 ± 69.07	227.44 ± 59.31	0.031
CRP (mg/L), mean ± SD	6.33 ± 12.34	4.06 ± 7.80	0.015
**Derived parameters—DAY 0**			
NLR 0	2.56 ± 1.45	2.47 ± 1.30	0.628
PLR 0	109.87 ± 46.38	120.36 ± 52.98	0.026
PWR 0	27.69 ± 7.50	31.32 ± 9.88	< 0.001

Legend: CRP—C-reactive protein, *n*—number of patients, NLR—neutrophil-to-lymphocyte ratio, PLR—platelet-to-lymphocyte ratio, PWR—platelet-to-WBC ratio, SD—standard deviation ratio, WBC—white blood cell count.

**Table 5:** Mean levels of post-operative inflammatory markers and derived parameters for patients with and without POD after CABG. POD, Postoperative delirium; CABG, coronary artery bypass grafting.

	CABG		
	Delirium + (*n* = 129)	Delirium − (*n* = 839)	*p*-Value
**Inflammatory parameters—DAY 1**			
Total WBC count (×10^9^/L), mean ± SD	11.52 ± 3.90	10.87 ± 3.47	0.096
Lymphocyte count (×10^9^/L), mean ± SD	1.17 ± 1.96	0.94 ± 0.80	0.646
Neutrophil count (×10^9^/L), mean ± SD	9.23 ± 3.03	8.84 ± 2.84	0.189
Platelets (×10^9^/L), mean ± SD	168.56 ± 63.38	176.63 ± 49.75	0.019
CRP (mg/L), mean ± SD	70.26 ± 34.39	65.63 ± 32.12	0.179
**Derived parameters—DAY 1**			
NLR 1	11.81 ± 6.82	11.32 ± 6.13	0.666
PLR 1	212.59 ± 119.03	226.55 ± 118.18	0.129
PWR 1	15.23 ± 5.21	17.36 ± 7.04	<0.001
**Inflammatory parameters—DAY 3**			
Total WBC count (×10^9^/L), mean ± SD	11.04 ± 3.18	10.60 ± 3.22	0.073
Lymphocyte count (×10^9^/L), mean ± SD	1.44 ± 1.21	1.43 ± 0.57	0.012
Neutrophil count (×10^9^/L), mean ± SD	18.37 ± 33.52	14.55 ± 32.51	0.002
Platelets (×10^9^/L), mean ± SD	155.50 ± 51.77	169.74 ± 50.68	0.001
CRP (mg/L), mean ± SD	248.13 ± 68.45	241.61 ± 70.95	0.234
**Derived parameters—DAY 3**			
NLR 3	7.52 ± 4.40	6.40 ± 3,45	0.002
PLR 3	130.97 ± 60.74	132.93 ± 62,08	0.963
PWR 3	14.71 ± 4.98	17.28 ± 8,07	<0.001
**Inflammatory parameters—DAY 5**			
Total WBC count (×10^9^/L), mean ± SD	9.55 ± 4.73	8.16 ± 2.64	<0.001
Lymphocyte count (×10^9^/L), mean ± SD	1.99 ± 3.08	1.85 ± 0.72	0.003
Neutrophil count (×10^9^/L), mean ± SD	6.40 ± 2.86	5.10 ± 2.30	<0.001
Platelets (×10^9^/L), mean ± SD	207.25 ± 72.43	227.80 ± 68.73	0,001
CRP (mg/L), mean ± SD	155.29 ± 73.56	122.09 ± 66.19	<0.001
**Derived parameters—DAY 5**			
NLR 5	4.68 ± 3.47	3.23 ± 2.51	<0.001
PLR 5	140.77 ± 66.14	135.71 ± 54.24	0.632
PWR 5	23.93 ± 10.57	29.87 ± 10.47	<0.001

Legend: CRP—C-reactive protein, *n*—number of patients, NLR—neutrophil-to-lymphocyte ratio, PLR—platelet-to-lymphocyte ratio, PWR—platelet-to-WBC ratio, SD—standard deviation ratio, WBC—white blood cell count.

**Table 6:** Exploratory multivariate logistic regression analysis for selected pre-operative parameters (Wald *p* < 0.05 threshold) predicting delirium after cardiac surgery.

Variables	OR	95 % CI	*p*-Value
Sex (male)	0.555	0.352–0.874	0.011
Age (years)	1.109	1.079–1.139	<0.001
NYHA grade	1.355	1.042–1.761	0.023
Extracardiac atherosclerosis	1.904	1.204–3.011	0.005
HbA1c (%)	1.330	1.120–1.579	<0.001
Platelet-to-WBC Ratio	0.947	0.922–0.972	<0.001

Legend: CI—confidence interval, HbA1c—glycated hemoglobin, NYHA—New York Heart Association, OR—odds ratio WBC—white blood cells.

**Table 7:** Reliable multivariate logistic regression analysis for the parameters most strongly associated (Wald *p* < 0.001) with POD after CABG. POD, Postoperative delirium; CABG, coronary artery bypass grafting.

Variable	OR	95 % CI	*p*-Value
Age (years)	1.114	1.084–1.144	**<0.001**
HbA1c (%)	1.406	1.191–1.660	**<0.001**
Platelet-to-WBC Ratio	0.952	0.928–0.976	**<0.001**

Legend: CI—confidence interval, HbA1c—glycated hemoglobin, OR—odds ratio, WBC—white blood cells.

**Table 8:** Receiver operating characteristic (ROC) analysis for objective preoperative factors used to determine postoperative delirium after CABG.

Predictor	AUC	Maximal Youden Index	Cut-Off Point	Sensitivity	Specificity	*p*-Value
**Age (years)**	0.712	0.34	67	0.752	0.585	<0.001
**HbA1c (%)**	0.598	0.16	6.40	0.395	0.764	<0.001
**PWR**	0.613	0.21	30.62	0.721	0.486	<0.001
**CARDEL Index**	0.742	0.37	8.26	0.612	0.753	<0.001

Legend: AUC—area under the curve, HbA1c—glycated hemoglobin, PWR—Platelet-to-WBC Ratio, CARDEL Index—CARdiac DELirium Index.

## References

[1] American Psychiatric Association (2013). Diagnostic and Statistical Manual of Mental Disorders.

[2] Barr J., Fraser G.L., Puntillo K., Ely E.W., Gélinas C., Dasta J.F., Davidson J.E., Devlin J.W., Kress J.P., Joffe A.M. (2013). American College of Critical Care Medicine. Clinical practice guidelines for the management of pain, agitation, and delirium in adult patients in the intensive care unit. Crit. Care Med..

[3] Ely E.W., Truman B., Speroff T., Harrell J.F.E., Dittus R.S., Shintani A., Gordon S.M., Inouye S.K., Bernard G.R. (2004). Delirium as a predictor of mortality in mechanically ventilated patients in the intensive care unit. JAMA.

[4] McPherson J.A., Wagner C.E., Boehm L.M., Hall J.D., Johnson D.C., Miller L.R., Burns K.M., Thompson J.L., Shintani A.K., Ely E.W. (2013). Delirium in the cardiovascular ICU. Crit. Care Med..

[5] Sockalingam S., Parekh N., Bogoch I.I., Sun J., Mahtani R., Beach C., Bollegalla N., Turzanski S., Seto E., Kim J. (2005). Delirium in the postoperative cardiac patient: A review. J. Card. Surg..

[6] Kotfis K., Marra A., Ely E.W. (2018). ICU delirium—A diagnostic and therapeutic challenge in the intensive care unit. Anaesthesiol. Intensive Ther..

[7] Kotfis K., Szylińska A., Listewnik M., Strzelbicka M., Brykczyński M., Rotter I., Zukowski M. (2018). Early delirium after cardiac surgery: An analysis of incidence and risk factors in elderly (≥65 years) and very elderly (≥80 years) patients. Clin. Interv. Aging.

[8] Girard T.D., Thompson J.L., Pandharipande P.P., E Brummel N., Jackson J.C., Patel M.B., Hughes C.G., Chandrasekhar R., Pun B.T., Boehm L.M. (2018). Clinical phenotypes of delirium during critical illness and severity of subsequent long-term cognitive impairment: A prospective cohort study. Lancet Respir. Med..

[9] Egberts A., Fekkes D., Wijnbeld E.H., Van Der Ploeg M.A., Van Saase J.L., Ziere G., Van Der Cammen T.J., Mattace-Raso F.U. (2015). Disturbed serotonergic neurotransmission and oxidative stress in elderly patients with delirium. Dement. Geriatr. Cogn. Dis. Extra..

[10] Maldonado J.R. (2013). Neuropathogenesis of delirium: Review of current etiologic theories and common pathways. Am. J. Geriatr. Psychiatry..

[11] Kotfis K., Zegan-Barańska M., Żukowski M., Kusza K., Kaczmarczyk M., Ely E.W. (2017). Multicenter assessment of sedation and delirium practices in the intensive care units in Poland—Is this common practice in Eastern Europe?. BMC Anesthesiol..

[12] Morandi A., Piva S., Ely E.W., Myatra S.N., Salluh J.I.F., Amare D., Azoulay E., Bellelli G., Csomos A., Fan E. (2017). Worldwide Survey of the “Assessing Pain, Both Spontaneous Awakening and Breathing Trials, Choice of Drugs, Delirium Monitoring/Management, Early Exercise/Mobility, and Family Empowerment” (ABCDEF) Bundle. Crit. Care Med..

[13] Khan B.A., Zawahiri M., Campbell N.L., Boustani M.A. (2011). Biomarkers for Delirium—A Review. J. Am. Geriatr. Soc..

[14] Androsova G., Krause R., Winterer G., Schneider R. (2015). Biomarkers of postoperative delirium and cognitive dysfunction. Front. Aging Neurosci..

[15] Egberts A., Wijnbeld E.H., Fekkes D., van der Ploeg M.A., Ziere G., Hooijkaas H., van der Cammen T.J., Mattace-Raso F.U. (2015). Neopterin: A potential biomarker for delirium in elderly patients. Dement. Geriatr. Cogn. Disord..

[16] Kulaksizoglu B., Kulaksizoglu S. (2016). Relationship between neutrophil/lymphocyte ratio with oxidative stress and psychopathology in patients with schizophrenia. Neuropsychiatr. Dis. Treat..

[17] Egberts A., Mattace-Raso F.U.S. (2017). Increased neutrophil-lymphocyte ratio in delirium: A pilot study. Clin. Interv. Aging.

[18] Shao Q., Chen K., Rha S.W., Lim H.E., Li G., Liu T. (2015). Usefulness of neutrophil/lymphocyte ratio as a predictor of atrial fibrillation: A meta-analysis. Arch. Med. Res..

[19] Wang X., Zhang G., Jiang X., Zhu H., Lu Z., Xu L. (2014). Neutrophil to lymphocyte ratio in relation to risk of all-cause mortality and cardiovascular events among patients undergoing angiography or cardiac revascularization: A meta-analysis of observational studies. Atherosclerosis.

[20] Yesil Y., Halil M., Ulger Z., Cankurtaran M., Kuyumcu M., Öztürk Z., Kizilarslanoğlu C., Etgül S., Arıoğul S. (2012). The evaluation of neutrophil-lymphocyte ratio in Alzheimer’s disease. Dement. Geriatr. Cogn. Disord..

[21] Gökhan S., Ozhasenekler A., Mansur Durgun H., Akil E., Ustündag M., Orak M. (2013). Neutrophil lymphocyte ratios in stroke subtypes and transient ischemic attack. Eur. Rev. Med. Pharmacol. Sci..

[22] Celikbilek M., Doğan S., Özbakir Ö., Zararsiz G., Küçük H., Gursoy S., Yurci A., Güven K., Yücesoy M. (2013). Neutrophil-lymphocyte ratio as a predictor of disease severity in ulcerative colitis. J. Clin. Lab. Anal..

[23] Lixiu L., Yuncheng X., Chunmei C., Ping C., Canhui P. (2015). Neutrophil-lymphocyte ratio in systemic lupus erythematosus disease: A retrospective study. Int. J. Clin. Exp. Med..

[24] Templeton A.J., McNamara M.G., Šeruga B., Vera-Badillo F.E., Aneja P., Ocaña A., Leibowitz-Amit R., Sonpavde G., Knox J.J., Tran B. (2014). Prognostic role of neutrophil-to-lymphocyte ratio in solid tumors: A systematic review and meta-analysis. J. Natl. Cancer Inst..

[25] Zadora P., Dabrowski W., Czarko K., Smoleń A., Kotlinska-Hasiec E., Wiorkowski K., Sikora A., Jarosz B., Kura K., Rola R. (2015). Preoperative neutrophil-lymphocyte count ratio heps predict the grade of glial tumor—A pilot study. Neurol. Neurochir. Pol..

[26] Qin B., Ma N., Tang Q., Wei T., Yang M., Fu H., Hu Z., Liang Y., Yang Z., Zhong R. (2016). Neutrophil lymphocyte ratio and platelet-lymphocyte ratio were useful markers in assessment of inflammatory response and disease activity in SLE patients. Mod. Rheumatol..

[27] Wu Y., Chen Y., Yang X., Chen L., Yang Y. (2016). Neutrophil-lymphocyte ratio and platelet-lymphocyte ratio were associated with disease activity in patients with SLE. Int. Immunopharmacol..

[28] De Jager C.P.C., Wever P.C., Gemen E.F.A., Kusters R., Van Gageldonk-Lafeber A.B., Van Der Poll T., Laheij R.J.F. (2012). The neutrophil-lymphocyte count ratio in patients with community-acquired pneumonia. PLoS ONE.

[29] Núñez J., Nunez E., Bodí V., Sanchis J., Minana G., Mainar L., Santas E., Merlos P., Rumiz E., Darmofal H. (2008). Usefulness of the neutrophil to lymphocyte ratio in predicting long-term mortality in ST segment elevation myocardial infarction. Am. J. Cardiol..

[30] Inoue S., Vasilevskis E.E., Pandharipande P.P., Girard T.D., Graves A.J., Thompson J., Shintani A., Ely E.W. (2015). The impact of lymphopenia on delirium in ICU patients. PLoS ONE.

[31] MacLullich A.M., Edelshain B.T., Hall R.J., De Vries A., Howie S.E., Pearson A., Middleton S.D., Gillies F., Armstrong I.R., White T.O. (2011). Cerebrospinal fluid interleukin-8 levels are higher in people with hip fracture with perioperative delirium than in controls. J. Am. Geriatr. Soc..

[32] Summers C., Rankin S.M., Condliffe A.M., Singh N., Peters A.M., Chilvers E.R. (2010). Neutrophil kinetics in health and disease. Trends Immunol..

[33] Zahorec R. (2001). Ratio of neutrophil to lymphocyte counts—Rapid and simple parameter of systemic inflammation and stress in critically ill. Bratisl. Lek. Listy..

[34] Kotfis K., Biernawska J., Zegan-Barańska M., Żukowski M. (2015). Peripheral Blood Lymphocyte Subsets (CD4+, CD8+ T Cells, NK Cells) in Patients with Cardiovascular and Neurological Complications after Carotid Endarterectomy. Int. J. Mol. Sci..

[35] Simone M.J., Tan Z.S. (2011). The role of inflammation in the pathogenesis of delirium and dementia in older adults: A review. CNS Neurosci. Ther..

[36] Jie Y., Gong J., Xiao C., Zhu S., Zhou W., Luo J., Chong Y., Hu B. (2018). Low Platelet to White Blood Cell Ratio Indicates Poor Prognosis for Acute-On-Chronic Liver Failure. BioMed Res. Int..

[37] Turkmen K., Erdur F.M., Ozcicek F., Ozcicek A., Akbaş E.M., Ozbicer A., Demirtas L., Turk S., Tonbul H.Z., Ozbıcer A. (2013). Platelet-to-lymphocyte ratio better predicts inflammation than neutrophil-to-lymphocyte ratio in endstage renal disease patients. Hemodial. Int..

[38] Chen Z., Huang Y., Li S., Lin J., Liu W., Ding Z., Li X., Chen Y., Pang W., Yang D. (2016). Platelet-to-White Blood Cell Ratio: A Prognostic Predictor for 90-Day Outcomes in Ischemic Stroke Patients with Intravenous Thrombolysis. J. Stroke Cerebrovasc. Dis..

[39] Garbens A., Wallis C.J., Bjarnason G., Kulkarni G.S., Nathens A.B., Nam R.K., Satkunasivam R. (2017). Platelet to white blood cell ratio predicts 30-day postoperative infectious complications in patients undergoing radical nephrectomy for renal malignancy. Can. Urol. Assoc. J..

[40] Kotfis K., Bott-Olejnik M., Szylińska A., Rotter I. (2019). Could Neutrophil-to-Lymphocyte Ratio (NLR) Serve as a Potential Marker for Delirium Prediction in Patients with Acute Ischemic Stroke? A Prospective Observational Study. J. Clin. Med..

[41] Dhabhar F.S., Malarkey W.B., Neri E., McEwen B.S. (2012). Stress-induced redistribution of immune cells—From barracks to boulevards to battlefields: A tale of three hormones—Curt Richter Award winner. Psychoneuroendocrinology.

[42] Kolaczkowska E., Kubes P. (2013). Neutrophil recruitment and function in health and inflammation. Nat. Rev. Immunol..

[43] Lindroth H., Bratzke L., Purvis S., Brown R., Coburn M., Mrkobrada M., Chan M.T.V., Davis D.H.J., Pandharipande P., Carlsson C.M. (2018). Systematic review of prediction models for delirium in the older adult inpatient. BMJ Open.

